# Reduction of Crosstalk Errors in a Surface Encoder Having a Long *Z*-Directional Measuring Range

**DOI:** 10.3390/s22239563

**Published:** 2022-12-06

**Authors:** Yifan Hong, Ryo Sato, Yuki Shimizu, Hiraku Matsukuma, Hiroki Shimizu, Wei Gao

**Affiliations:** 1 Precision Nanometrology Laboratory, Department of Finemechanics, Tohoku University, Sendai 980-8579, Japan; 2 Division of Mechanical and Space Engineering, Graduate School of Engineering, Hokkaido University, Kita 13, Nishi 8, Kita-ku, Sapporo 060-8628, Japan; 3 Department of Engineering, Graduate School of Engineering, Kyushu Institute of Technology, 1-1 Sensui-cho, Tobata-ku, Kitakyushu-shi, Fukuoka 804-8550, Japan

**Keywords:** reduction of crosstalk errors, multi-axis measurement, surface encoder, *Z*-directional measuring range, Michelson-type interferometer

## Abstract

A modified two-axis surface encoder is proposed to separately measure both the in-plane displacement and the *Z*-directional out-of-plane displacement with minor crosstalk errors. The surface encoder is composed of a scale grating and a small-sized sensor head. In the modified surface encoder, the measurement laser beam from the sensor head is designed to be projected onto the scale grating at a right angle. For measurement of the *X*- and *Y*-directional in-plane scale displacement, the positive and negative first-order diffracted beams from the scale grating are superimposed on each other in the sensor head, producing interference signals. On the other hand, the *Z*-directional out-of-plane scale displacement is measured based on the principle of a Michelson-type interferometer. To avoid the influence of reflection from the middle area of the transparent grating, which causes periodic crosstalk errors in the previous research, a specially fabricated transparent grating with a hole in the middle is employed in the newly designed optical system. A prototype sensor head is constructed, and basic performances of the modified surface encoder are tested by experiments.

## 1. Introduction

Precision positioning is a fundamental operation in manufacturing, such as tool positioning with respect to a workpiece in a machine tool or the positioning of a probe with respect to a measurement target in a profile measuring instrument [1,2,3,4]. As the basis for precision positioning, displacement sensors for single-axis measurement, including laser interferometers [5,6] and linear encoders [7,8] have been developed over the past several decades. The resolution of those sensors has been proven to achieve sub-nanometric accuracy. As the increase of product performance demands, component structures become increasingly delicate and complex, often requiring machining in multiple directions [9]. At the same time, the need to measure the geometry of products in all directions is also increasing on a daily basis [10]. Multi-axis measuring systems based on the multi-axis polar coordinate system, the multi-axis Cartesian coordinate system, the triangulation system, or the multilateration system have therefore been developed so far for multi-axis precision positioning [11,12].

Among these coordinate systems, the Cartesian coordinate system is most commonly used in applications requiring high positioning accuracy [5,12]. Most of the measuring instruments based on multi-axis Cartesian coordinate systems, such as Coordinate Measuring Machines (CMMs), employ multiple sensors in mutually orthogonal directions [13,14]. In these applications, misalignment of each sensor could result in abbe errors [15,16]. The accumulation of these errors could lead to a decrease in measurement accuracy. Although it is possible to avoid abbe errors in a laser interferometric system by carefully aligning a laser beam in each axis [17,18], it is time consuming. Additionally, the whole measurement system tends to be large and complex. Furthermore, to maintain the accuracy of laser interferometers, the ambient environment such as the temperature, humidity, and air pressure should be controlled strictly [19,20,21,22]. Therefore, a multi-axis measurement system complying with Abbe’s principle designed in a compact size and less susceptible to the surrounding environment is required.

Planar encoders have been proposed to detect two-axis in-plane displacements [23,24,25]. To also detect the out-of-plane displacement, the authors’ group have been working on the development of a three-axis displacement sensor referred to as the surface encoder [26,27,28]. The three-axis displacements can be detected from the physical gradations of the scale grating and wavelength benchmark, which are read by the sensor head. Since the three-axis displacements are measured by a single measurement laser beam irradiated onto the scale grating at a point, the entire system is simple, and the abbe errors can be avoided. Moreover, the surface encoder is more robust against environmental disturbances than the conventional interferometer-based multi-axis measuring system due to its shorter optical path length between the scale grating and sensor head. The in-plane measuring area of the surface encoder is proportional to the size of the scale grating, and it can be easily extended with the employment of the mosaic scale grating technique [29,30,31]. Meanwhile, the measuring range for the *Z*-directional out-of-plane displacement is restricted to approximately ±150 μm due to the nature of its design; the position of the first-order diffracted beams shift as the scale grating moves along the *Z*-direction, making it difficult to superimpose the diffracted beams on reference beams and preventing the generation of interference signals. The usage of the surface encoder has thus been restricted to the applications such as the feedback control of a surface motor-driven planar stage [32] where the shorter *Z*-directional measuring range can be accepted. Although other groups have also proposed some multi-axis encoders [33,34] that can measure out-of-plane displacement in millimeters, their systems tend to be large.

To overcome the above-mentioned drawbacks of the conventional multi-axis optical encoders, the authors have proposed a new small-sized optical surface encoder [35]. With the designed optical surface encoder, a *Z*-directional measuring range of ±1.5 mm has been successfully achieved. Additionally, the optical sensor head has been designed in a compact size of 110 mm (*X*) × 115 mm (*Z*) × 40 mm (*Y*). However, unexpected periodic crosstalk errors in the interference signals have been found to degrade the measurement accuracy of the surface encoder with the new optical configuration. To address the issue, in this paper, theoretical analysis is carried out to confirm the causes of periodic crosstalk errors in the surface encoder. After that, an optimized optical configuration is proposed for the surface encoder. The basic principle of the optimized surface encoder is explained, and some experiments are carried out using the newly developed surface encoder with the optimized optical configuration.

## 2. Theoretical Approach

**Figure 1:**
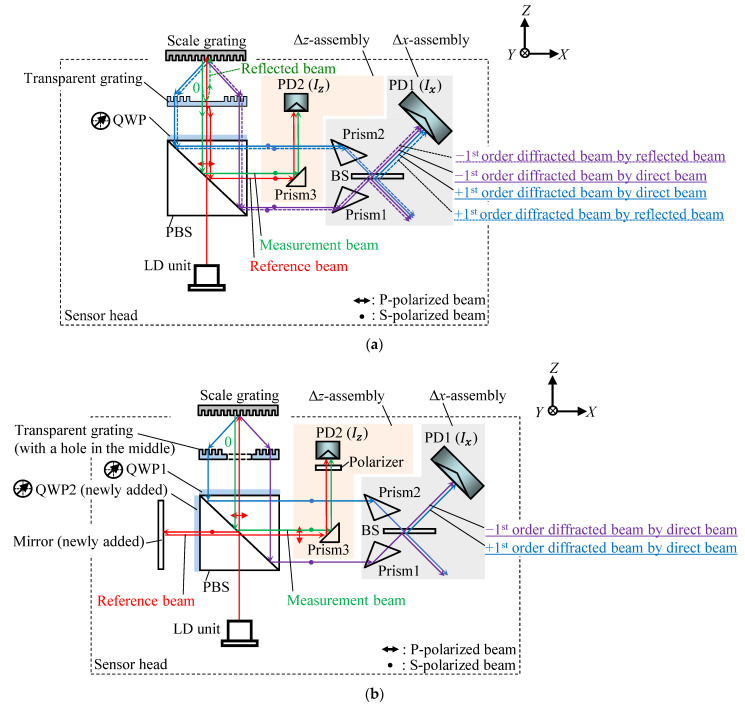
Optical configurations of the surface encoder: (**a**) The optical configuration of the conventional surface encoder with an expanded Z-directional measuring range; (**b**) The optical configuration of the newly proposed surface encoder.

Figure 1a shows a schematic of the surface encoder with a long *Z*-directional measuring range proposed in the previous study [35] by the authors’ group. The optical sensor head of the surface encoder consists of a Δ*x*-assembly and a Δ*z*-assembly, which are used for measuring the scale grating’s *X*- and Z-directional displacements, respectively. In the Δ*x*-assembly, the interference signal *I_x_* generated from the positive and negative first-order diffracted beams presented, is detected by a photodetector (PD1). Similarly, the interference signal *I_z_* generated by superimposing the reference beam reflected from the transparent grating and the zeroth-order diffracted beam from the scale grating is detected by another photodiode (PD2) in the Δ*z*-assembly. The *X*- and *Z*-directional displacements can be calculated by the interference signal *I_x_* and *I_z_*, respectively. As is shown by the dotted lines in Figure 1a, a part of the zeroth-order diffracted beam from the scale grating is reflected by the surface of the transparent grating and projected onto the scale grating again, producing diffracted beams. This results in a periodic crosstalk signal *I_x_*(Δ*z*) associated with the *Z*-directional scale grating displacement. When the scale grating travels along the *Z*-axis, the electric fields of the positive and negative first-order diffracted beams produced by the direct beam *E*_±1_(Δ*z*) and those produced by the reflected zeroth-order diffracted beam *E*_(0,±1)_(Δ*z*) are expressed by the following equations:

(1) $$E_{\pm 1}\left(\Delta z\right)=A_{\pm 1}\exp\left(i\varphi_{\pm 1}\left(\Delta z\right)\right)$$

(2) $$E_{\left(0,\pm 1\right)}\left(\Delta z\right)=A_{\left(0,\pm 1\right)}\exp\left(i\varphi_{\left(0,\pm 1\right)}\left(\Delta z\right)\right)$$

where *A*_±1_, *A*_(0,±1)_ are the complex amplitudes; *φ*_±1_(Δ*z*), *φ*_(0,±1)_(Δ*z*) are the phase shifts of the beams corresponding to the *Z*-directional scale grating displacement ∆*z*, and they can be calculated as follows:

(3) $$\varphi_{\pm 1}\left(\Delta z\right)=2\pi \frac{\left(1+\cos\theta \right)\Delta z}{\lambda }$$

(4) $$\varphi_{\left(0,\pm 1\right)}\left(\Delta z\right)=2\pi \frac{\left(3+\cos\theta \right)\Delta z}{\lambda }$$

where *θ* is the angle of diffraction of the first-order diffracted beams, and *λ* is the wavelength of the laser beam. Based on the theory of multi-beam interference, the electric field of the crosstalk signal produced by the interference of the above first-order diffracted beams can thus be calculated as the following equation:

(5) $$E_{x}\left(\Delta z\right)=E_{+1}\left(\Delta z\right)+E_{-1}\left(\Delta z\right)+E_{\left(0,+1\right)}\left(\Delta z\right)+E_{\left(0,-1\right)}\left(\Delta z\right)$$

As a result, the intensity of the crosstalk signal *I_x_*(Δ*z*) detected by PD1 can be obtained as a conjugate complex multiplication of *E_x_*(Δ*z*) as follows:

(6) $$\begin{array}{l} I_{x}\left(\Delta z\right)=\frac{1}{2}E_{x}\left(\Delta z\right)\cdot \bar{E_{x}\left(\Delta z\right)}\\ \;\;\;=\frac{1}{2}\left(A_{+1}^{2}+A_{-1}^{2}+A_{\left(0,+1\right)}^{2}+A_{\left(0,-1\right)}^{2}\right)+\left(A_{+1}A_{\left(0,+1\right)}+A_{-1}A_{\left(0,-1\right)}\right)\cos\left(4\pi \frac{\Delta z}{\lambda }\right) \end{array}$$

From the above equation, the period of the crosstalk signal *I_x_*(Δ*z*) is found to be *λ*/2, which is consistent with the experimental results in the previous study [35]. 

To avoid the influence of reflection from the middle area of the transparent grating used in the previous study, the proposed method employs a different transparent grating having a hole in the middle so that the zeroth-order diffracted beam will directly pass through the transparent grating as shown in Figure 1b; this contributes to prevent the generation of diffracted beams by the reflected beam. It should be noted that a transparent grating coated with an anti-reflective film or a prism unit [22,28] can also be employed for this purpose. Due to the modification on the transparent gratings, a different principle is employed for measurement of the *Z*-directional displacement of the scale grating. To generate the reference beam, a mirror and another quarter wave plate (QWP2) are added to the optical head; namely, the *Z*-directional displacement measurement is carried out based on the Michelson-type interferometer. The proposed optical configuration is expected to reduce the periodic crosstalk error component in *I_x_*(Δ*z*), which is the purpose of the modification of the optical head in this paper.

**Figure 2:**
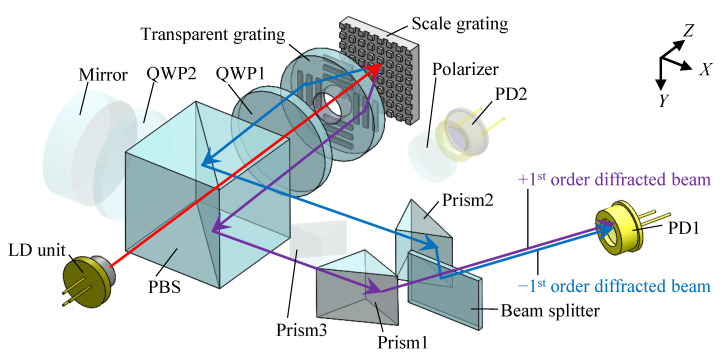
Optical system related to the *X*-directional displacement measurement (the transparency of the unrelated components is increased).

The principle of the in-plane displacement measurement with the proposed optical configuration is the same as the conventional surface encoder [35]. Figure 2 shows a schematic of the proposed optical configuration regarding the *X*-directional displacement measurement. A collimated laser beam from a laser diode (LD) unit is at first divided into P- and S-polarized beams by a polarizing beam splitter (PBS). The P-polarized beam passes through the PBS and is circularly polarized by a quarter wave plate (QWP). Since there is a hole in the middle of the transparent grating, the circularly polarized beam can be projected onto the scale grating directly, producing positive and negative first-order diffracted beams. The produced first-order diffracted beams are aligned to be parallel with each other by the transparent grating having four areas with one-axis grating pattern structures whose periods are the same as the scale grating. The paralleled first-order diffracted beams pass through the QWP again to become S-polarized beams. After being reflected at the PBS, the positive and negative first-order diffracted beams are reflected at prism1 and prism2, respectively. Then, the first-order diffracted beams are superimposed on a beam splitter (BS), generating an interference signal. The normalized intensity of the interference signal *I_x_* observed by a photodiode (PD1) can be described as follows:

(7) $$I_{x}=\cos\left(\frac{4\pi }{g}\Delta x\right)$$

in the above equation, the parameter *g* is the grating period. The *X*-directional scale displacement Δ*x* can thus be obtained as follows:

(8) $$\Delta x=\left(\cos^{-1}I_{x}\right)\frac{g}{4\pi }$$

It should be noted that the diffracted beams are superimposed no matter how the beams shift as the scale grating travels along the *Z*-direction since prism1 and prism2 are placed symmetrically with respect to the BS. By applying such an optical system, an expanded measuring range for *Z*-axis measurement can be achieved.

**Figure 3:**
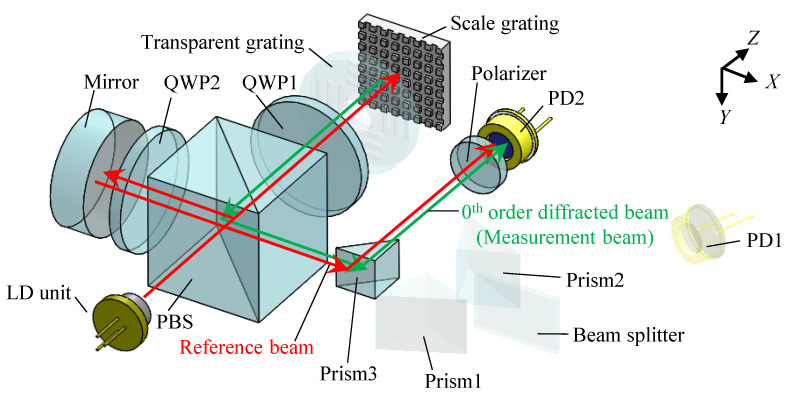
Optical system related to the *Z*-directional displacement measurement (the transparency of the unrelated components is increased).

Measurement of the *Z*-directional displacement is carried out based on the Michelson-type interferometer. Figure 3 shows the optical components in the proposed optical configuration related to the *Z*-directional displacement measurement. The reference beam from a mirror and the zeroth-order diffracted beam from the scale grating are superimposed and reflected at prism3 to generate an interference signal. It should be noted that a polarizer is employed for constructive interference between the two beams. Another photodiode (PD2) is employed to observe the interference signal. The normalized intensity of the interference signal *I_z_* can be expressed as follows:

(9) $$I_{z}=\cos\left(\frac{4\pi }{\lambda }\Delta z\right)$$

Then, the *Z*-directional scale displacement Δ*z* can be obtained as follows:

(10) $$\Delta z=\left(\cos^{-1}I_{z}\right)\frac{\lambda }{4\pi }$$

## 3. Testing of the Modified Two-Axis Surface Encoder

**Figure 4:**
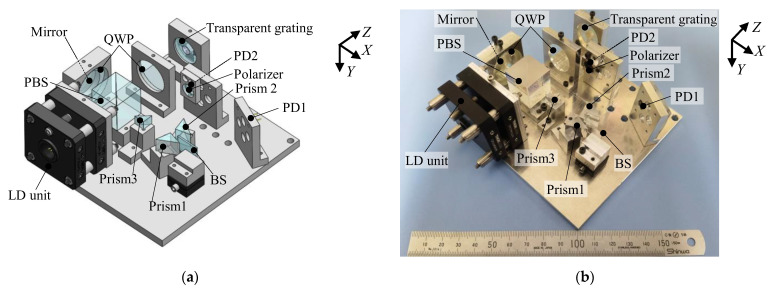
Prototype sensor head: (**a**) A three-dimensional model; (**b**) A photograph.

To verify the feasibility of the proposed optical configuration of the surface encoder, a prototype optical sensor head was designed and developed. A photograph of the developed optical sensor head is shown in Figure 4. The LD unit was composed of a laser diode (HL6756MG, Thorlabs, Newton, NJ, USA) with a wavelength of 670 nm, a collimating lens (A240TM-A, Thorlabs, Japan) for collimating the emitted beam, and an aperture (16-829, Edmund, Japan) for making the beam diameter to be 1 mm. A PBS (PBS-20-6700, Sigmakoki, Japan) with dimensions of 20 mm × 20 mm × 20 mm was placed right after the LD unit. Two QWPs (WPQ10E-670, Thorlabs, Japan) were coated with anti-reflective films to reduce reflection, and the fast axes of QWPs were set to be 45 degrees with respect to the direction of the P-polarized beam. A dual-beam interferometer was used to fabricate the transparent grating using interference lithography [36,37,38]. To avoid a large sensor head, a plate-type BS (BSS10R, Thorlabs, Japan) was employed instead of a cube-type BS. Considering the beam shift when scale grating travels along the *Z*-axis, the dimensions of prism1 and prism2 were chosen to be 10 mm × 10 mm × 10 mm (RPB2-10-550, Sigmakoki, Japan). It should be noted that prism3 should be small so that it will not block the first-order diffracted beams; therefore, its dimensions were chosen to be 7 mm × 7 mm × 7 mm (RPB3-07-550, Sigmakoki, Japan). The size of the optical sensor head remained the same as the conventional one: 110 mm (*X*) ×115 mm (*Z*) × 40 mm (*Y*).

**Figure 5:**
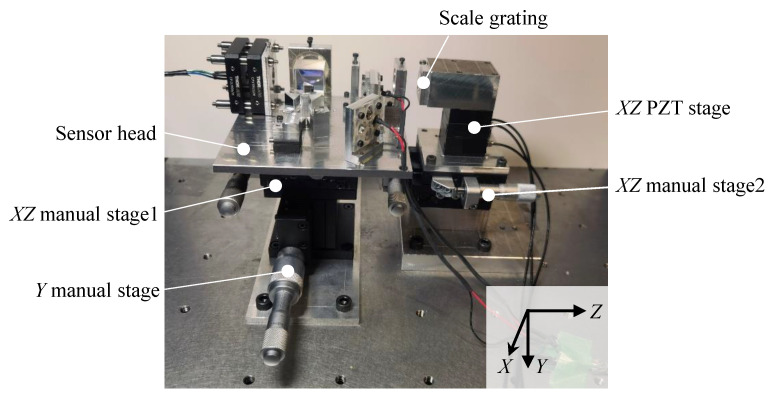
A photograph of the measurement setup.

Figure 5 shows a photograph of the constructed experimental setup. The planar position and vertical height of the sensor head relative to the scale grating should be adjusted so that the incident beam could be projected onto the middle of the scale grating. The distance between the sensor head and the scale grating was adjusted to be 9 mm. For adjusting the planar position, *XZ* manual stage1 and 2 (TSD-602SDM, Sigmakoki, Japan) were employed, and the sensor head and the scale grating were mounted on these stages. Meanwhile, a *Y* manual stage (LV-642-1, Chuo Precision Industrial, Tokyo, Japan) was used to adjust the vertical height of the sensor head. Additionally, an *XZ* PZT stage was employed in the experiment to give displacement commands along the *X*- and *Z*-axes. The travel range of the PZT stage was 50 μm, and the closed loop resolution in each axis was 0.2 nm.

By using the constructed experimental setup, the basic performances of the proposed surface encoder were tested. At first, the interference signals obtained by the conventional surface encoder and the newly developed one were compared. The scale gratings of the surface encoders were moved along the *X*- and *Z*-axes, respectively, by the PZT stage at the same fixed speed of 500 nm/s. 

**Figure 6:**
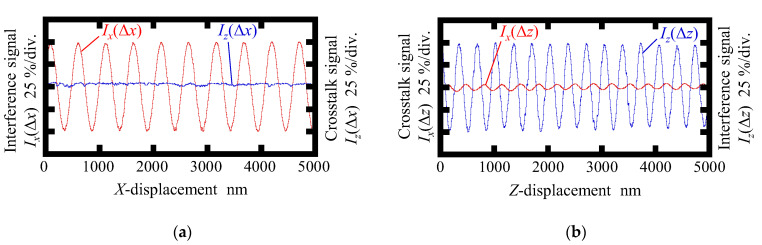
Interference signal and corresponding crosstalk signal observed in the conventional surface encoder: (**a**) Readouts of *I_x_* and *I_z_* when giving the *X*-displacement; (**b**) Readouts of *I_x_* and *I_z_* when giving the *Z*-displacement.

**Figure 7:**
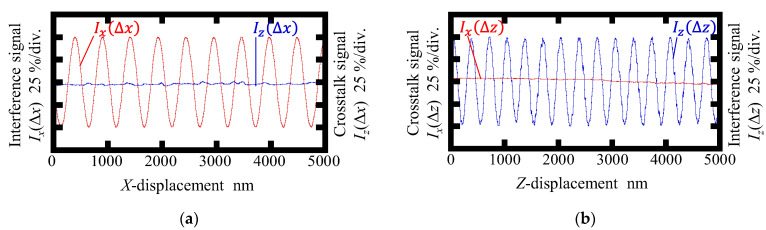
Interference signal and corresponding crosstalk signal observed in the proposed surface encoder: (**a**) Readouts of *I_x_* and *I_z_* when giving the *X*-displacement; (**b**) Readouts of *I_x_* and *I_z_* when giving the *Z*-displacement.

Figure 6 and Figure 7 show the interference signal and corresponding crosstalk signal observed in the conventional surface encoder and the newly proposed one, respectively. As can be seen in the figures, the interference signals *I_x_*(Δ*x*) and *I_z_*(Δ*z*) were successfully obtained in both encoders. The amplitude of the crosstalk signal *I_z_*(Δ*x*) was found to be small, as shown in Figure 6a, indicating that the interference signal *I_z_* was independent of the *X*-directional scale displacement. However, as can be seen in Figure 6b, a periodic change was found in *I_x_*(Δ*z*) in the conventional surface encoder; the period was found to be almost the same as that of the interference signal *I_z_*(Δ*z*). On the other hand, as can be seen in Figure 7, the periodic component of the crosstalk signal *I_x_*(Δ*z*) is eliminated successfully in the newly proposed surface encoder. 

**Figure 8:**
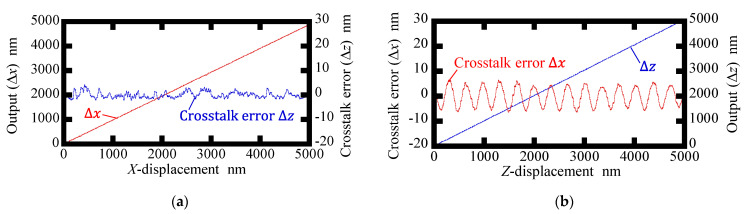
Crosstalk errors observed in the conventional surface encoder: (**a**) Readouts of Δ*x* and Δ*z* when giving the *X*-displacement; (**b**) Readouts of Δ*x* and Δ*z* when giving the *Z*-displacement.

**Figure 9:**
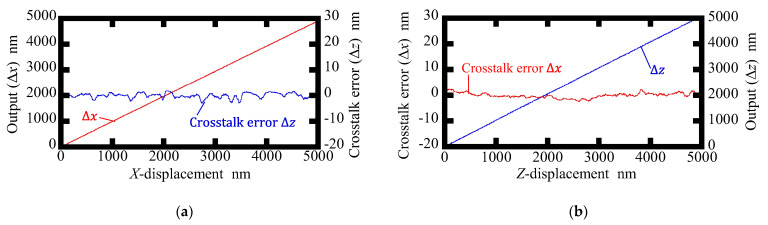
Crosstalk errors observed in the proposed surface encoder: (**a**) Readouts of Δ*x* and Δ*z* when giving the *X*-displacement; (**b**) Readouts of Δ*x* and Δ*z* when giving the *Z*-displacement.

Figure 8 and Figure 9 show displacement outputs and crosstalk errors of the conventional surface encoder and the newly proposed one, respectively. Amplitudes of the crosstalk errors in the conventional surface encoder were found to be approximately 4.0 nm and 6.5 nm for Δ*z* and Δ*x*, respectively, while those in the proposed surface encoder were found to be reduced to approximately 4.0 nm and 2.0 nm for Δ*z* and Δ*x*, respectively. It should be noted that the linear components of the crosstalk errors mainly caused by the misalignment between the sensor head and the axes of the scale grating are removed in Figure 8 and Figure 9. A more careful alignment of the setup is expected to reduce the linear components.

**Figure 10:**
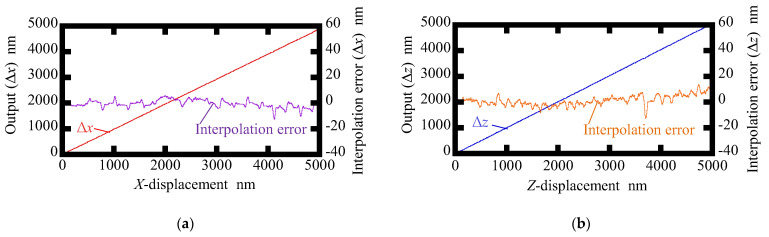
Interpolation errors of the proposed method of this research: (**a**) Interpolation error in *X*-axis displacement measurement; (**b**) Interpolation error in *Z*-axis displacement measurement.

To further verify the feasibility of the proposed surface encoder, interpolation errors in each axis were investigated. In this experiment, the scale grating was made to travel along the *X*- or *Z*-axis at a fixed speed of 500 nm/s by using the PZT stage. The interpolation errors when measuring the *X*- and *Z*-directional scale displacements are shown in Figure 10a and Figure 10b, respectively. The interpolation errors were found to be approximately ±8 nm and ±14 nm for the measurement of the *X*- and *Z*-displacements, respectively. Imperfections in the optical components, misalignment between axes of components, and nonlinearities in signal processing systems could result in interpolation errors [39,40].

**Figure 11:**
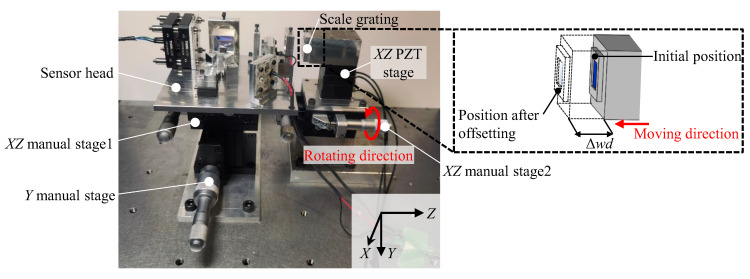
Schematic of the experimental setup when giving the offset to test the *Z*-directional measurement range of the surface encoder.

Experiments were expanded to confirm the measuring range of the proposed surface encoder along the *Z*-axis. Figure 11 shows the experimental setup. At the initial setup, the gap between the scale and the sensor head was set to be 9 mm. A *Z*-directional offset Δ*wd* of 1 mm step was then applied to the scale grating by *XZ* manual stage2. At every position of the scale grating, the basic performances of the surface encoder were tested. 

**Figure 12:**
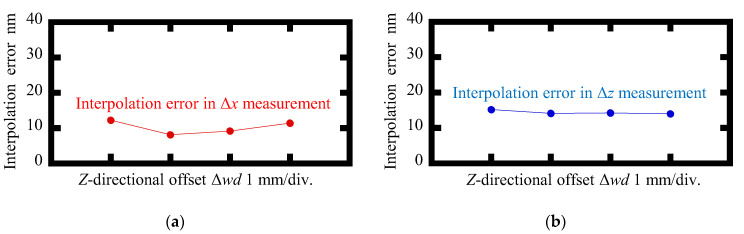
Variations of amplitudes of the interpolation errors when the *Z*-directional offset Δ*wd* was applied to the scale grating: (**a**) Δ*x* measurement; (**b**) Δ*z* measurement.

**Figure 13:**
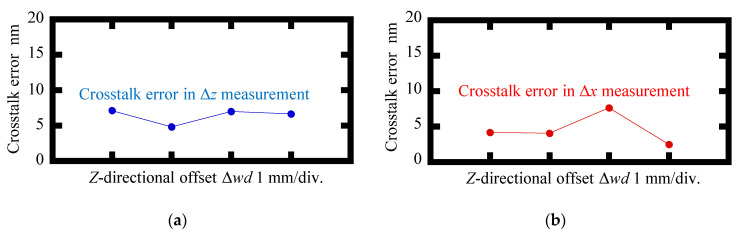
Variations in amplitudes of the crosstalk errors when the *Z*-directional offset Δ*wd* was applied to the scale grating: (**a**) Readout of Δ*z* when *X*-axis displacement was applied to the scale grating; (**b**) Readout of Δ*x* when *Z*-axis displacement was applied to the scale grating.

Figure 12 and Figure 13 show the variations of the amplitudes of the interpolation errors and the crosstalk errors of the proposed surface encoder, respectively, when giving the *Z*-directional offset to the scale grating. No significant changes in the interpolation errors and crosstalk errors were found in an offset range of 3 mm. These results demonstrated that the proposed surface encoder achieved a *Z*-directional measuring range of at least ±1.5 mm. 

The above experimental results show that the proposed surface encoder can measure not only in-plane displacement, but also out-of-plane displacement compared with planar encoders [23,24,25]. Additionally, it expanded its measuring range in out-of-plane direction (*Z*-axis direction) from hundreds of micrometers to ±1.5 mm compared with conventional surface encoders [26,27,28] while reducing the optical size [33,34]. The crosstalk errors observed in the previous study [35], which is the main focus of this paper, are reduced to ±2 nm and ±4 nm in Δ*x* and Δ*z* measurements.

## 4. Conclusions

A new optical configuration has been proposed for the surface encoder, which can measure the in-plane displacement (*X*-axis) and long-range out-of-plane (*Z*-axis) displacement simultaneously. At first, the causes of the periodic crosstalk error for *X*-axis displacement measurement in the previous research have been investigated, and the influence of the reflected laser beam from the middle area of the transparent grating has been confirmed to be the main cause of the periodic crosstalk error. A specially fabricated transparent grating with a hole in the middle has thus been employed to avoid the influence of reflection from the surface of the middle area of the transparent grating. Additionally, a mirror has been employed to generate a reference beam; namely, the principle of the *Z*-directional displacement measurement has been switched from the Fizeau-type interferometer to the Michelson-type interferometer. A prototype surface encoder and the experimental setup have been constructed. The optical sensor head has been designed in the size of 110 mm (*X*) × 115 mm (*Z*) × 40 mm (*Y*). The periodic components of the crosstalk signal have been eliminated in the newly proposed surface encoder, and the crosstalk errors have been reduced to ±2 nm and ±4 nm in Δ*x* and Δ*z* measurements. Other basic performances of the proposed surface encoder have also been tested. The readouts of the surface encoder have shown good linearity with respect to the input displacement, with interpolation errors of ±8 nm and ±14 nm in Δ*x* and Δ*z* measurements. The *Z*-directional measuring range of the proposed surface encoder has been confirmed to be at least ±1.5 mm. With the expanded *Z*-directional measuring range, the proposed surface encoder can be applied for precision positioning of multi-axis stages and multi-axis measuring machines and so forth, in which the conventional surface encoder could not have been applied due to the limited *Z*-directional measuring range. 

It should be noted that, focusing on the modified principle of the extended *Z*-directional measurement mechanism, the surface encoder has been designed to measure the uni-directional displacements in the *X*- and *Z*-axes. The modification of the optical system to realize the bi-directional displacement measurements along the three (*X*-, *Y*-, *Z*-) axes will be carried out as future work. Although the proposed surface encoder in this paper can only be used for two-axis displacement measurement, it can easily be extended to three-axis displacement measurement by adding the function of the *Y*-axis displacement measurement. For this purpose, the *Y*-directional positive and negative first-order diffracted beams should be utilized, and a Δ*y*-assembly identical to the Δ*x*-assembly should be added to the system. Future work also includes further experimental investigation for more accurate performance assessment of the proposed surface encoder. 

## Data Availability

The data presented in this study are available on request from the corresponding author.
